# CyNEST: a maintainable Cython-based interface for the NEST simulator

**DOI:** 10.3389/fninf.2014.00023

**Published:** 2014-03-14

**Authors:** Yury V. Zaytsev, Abigail Morrison

**Affiliations:** ^1^Simulation Laboratory Neuroscience – Bernstein Facility for Simulation and Database Technology, Institute for Advanced Simulation, Jülich Aachen Research Alliance, Jülich Research CenterJülich, Germany; ^2^Faculty of Biology, Albert-Ludwig University of FreiburgFreiburg im Breisgau, Germany; ^3^Institute for Advanced Simulation (IAS-6), Theoretical Neuroscience and Institute of Neuroscience and Medicine (INM-6), Computational and Systems Neuroscience, Jülich Research Center and JARAJülich, Germany; ^4^Institute of Cognitive Neuroscience, Faculty of Psychology, Ruhr-University BochumBochum, Germany

**Keywords:** Python language, neural simulator, maintainability, technical debt, HPC

## Abstract

NEST is a simulator for large-scale networks of spiking point neuron models (Gewaltig and Diesmann, 2007). Originally, simulations were controlled via the Simulation Language Interpreter (SLI), a built-in scripting facility implementing a language derived from PostScript (Adobe Systems, Inc., 1999). The introduction of PyNEST (Eppler et al., 2008), the Python interface for NEST, enabled users to control simulations using Python. As the majority of NEST users found PyNEST easier to use and to combine with other applications, it immediately displaced SLI as the default NEST interface. However, developing and maintaining PyNEST has become increasingly difficult over time. This is partly because adding new features requires writing low-level C++ code intermixed with calls to the Python/C API, which is unrewarding. Moreover, the Python/C API evolves with each new version of Python, which results in a proliferation of version-dependent code branches. In this contribution we present the re-implementation of PyNEST in the Cython language, a superset of Python that additionally supports the declaration of C/C++ types for variables and class attributes, and provides a convenient foreign function interface (FFI) for invoking C/C++ routines (Behnel et al., 2011). Code generation via Cython allows the production of smaller and more maintainable bindings, including increased compatibility with all supported Python releases without additional burden for NEST developers. Furthermore, this novel approach opens up the possibility to support alternative implementations of the Python language at no cost given a functional Cython back-end for the corresponding implementation, and also enables cross-compilation of Python bindings for embedded systems and supercomputers alike.

## 1. Introduction

Several projects in simulation have established themselves in the domain of neuroscience as long-term providers of tools aiming to supply the community with the simulation technology that users can rely upon for a particular level of modeling. These include STEPS (Hepburn et al., 2012) for stochastic simulation of reaction-diffusion systems in three dimensions, NEURON (Carnevale and Hines, 2006) for empirically-based simulations of neurons and networks of neurons, and NEST (Gewaltig and Diesmann, 2007) for large-scale networks of spiking point neuron models. This process of establishement occurs partially as a result of their respective maintainers' consistent efforts to ensure the quality and the sustainability of these software packages, and partially by virtue of the fact that as the projects reach a critical level of usage in the community, it creates a positive feedback loop which reinforces their acceptance. This “crystallization” around successful simulators, such as (but not limited to) those mentioned above, is fueled both by an increasing demand from the scientific community for stable, performant, accurate and re-usable tools, and likewise by large collaborative efforts to establish common simulation platforms in neuroscience, including BrainScaleS ^1^ and the Human Brain Project ^2^.

However, in order to serve the community interests, it is not enough to produce documented high-quality software packages that provide solid implementations of efficient and scalable simulation algorithms. An often overlooked notion is that the *software needs to evolve throughout its whole life*. Hence, a software tool that can be maintained with ease is preferable to an otherwise excellent, but completely unmaintainable software package.

This need for continuous evolution is known as the “Law of Continuing Change” in the software engineering literature (Lehman, 1984) and stems from a number of factors. One of the most important of these factors, particularly in the context of scientific software, is the continuous evolution of the users' needs (or developers' perception thereof). Conversely, even if we assume that the software specifications do not need to change throughout its lifecycle, developers still need to perform regular modifications of the software in order to fix discovered defects, and keep adapting it in response to the changes in the environment, such as newly released versions of the operating systems, system compilers, library dependencies, cutting-edge hardware, etc.

Unfortunately, these continuous adaptations result in an increase of the project's *technical debt* (Cunningham, 1992). Technical debt is an increasingly popular metaphor in the industry to describe the phenomenon in which accumulating unattended individual quality issues in large software systems ultimately cause severe degradation of the system as a whole. Technical debt denotes the amount of effort that would have to be invested to return the system to an acceptable quality level. The quality issues are not necessarily due to negligence or poor decision making, but can also originate from external constraints obtaining at the time a particular design decision was made. Therefore, focused maintenance needs to be performed in order to counteract this tendency and ensure that the software remains useful in the future. Critical maintenance tasks to ensure the durability of software projects can be considered as falling into the following classification structure, loosely based on Lientz and Swanson (1980):

1. *Homeostatic maintenance* aims at closing the gap between intended and actual software operation (fixing discovered issues, ensuring compatibility with newest versions of the dependencies such as operating systems, compilers, libraries etc.).
2. *Evolutionary maintenance* aims at closing the gap between current and desired software operation (developing new functionality and adding new features in response to changes in requirements).
3. *Counter-revolutionary maintenance* aims at closing the gap between previous and revised assumptions on environmental constraints and opportunities (paying back the technical debt).

Many large applications have bindings to dynamic languages, which can be used for scripting their behavior or interacting with other software. One important example of this is Python bindings, which are usually provided with simulators written in a different language such as C/C++. They exhibit a classic maintainability issue as discussed above: there are multiple implementations of Python language, and multiple versions of those implementations, each with their own C API.

One approach for simulator developers to deal with this problem would be to restrict themselves to supporting only one, or a small number of Python implementations/versions thereof. However, this reduces the usefulness of the application, especially in the case that users do not have this aspect under their own control (e.g., when the Python version is defined by the project policy or interoperability requirements). Another possibility would be to introduce conditional branches to account for the differences between Python implementations in the course of homeostatic maintenance. Unfortunately, the resulting spaghetti code ^3^ inflates the code base and increases the maintenance burden pro rata.

Here we present a third option: in the spirit of counter-revolutionary maintenance, we have chosen to rewrite the PyNEST low-level API, originally implemented in a mixture of C and C++, in a higher level language (Cython). In Section 2 we introduce the necessary concepts and methods and explain how we have applied them to the NEST code base. We then demonstrate in Section 3 that the resulting code is now independent of the Python/C API, since all hand-crafted C API calls have been removed. The size of the code base was reduced almost by half, and the bindings became more comprehensible and maintainable. At the same time, a performance assessment of the new implementation reveals no substantial degradation in terms of runtime and memory consumption. Finally, in Section 4 we discuss the applicability of this approach to other neuroinformatics software as well as available alternatives.

## 2. Methods and materials

Software packages for large-scale simulations generally have to fulfill two quintessential requirements: high performance and high scalability. In the domain of high performance computing (HPC), most hardware vendors choose to focus on supporting development environments primarily for Fortran and C/C++ programming languages. Specifically, this means that they provide compilers that are able to emit highly tuned machine code that yields maximum performance on their hardware. They also make optimized libraries available that support established parallel and distributed programming paradigms, such as OpenMP and MPI, as well as additional facilities to fully exploit the capabilities of the hardware, such as processor features (e.g., header files declaring intrinsic functions) or interconnect topology (including proprietary libraries or system calls). Consequently, if a simulation software is intended to extract the maximally achievable performance out of the available HPC hardware, the choices of programming languages to implement its core are essentially reduced to Fortran and C/C++.

Nevertheless, a productive user interface is also an important requirement for a successful simulator. This is especially true in the research context, where scientists' productivity is determined not only by the runtime of individual simulations, but also by the time required to evolve these simulations into follow-up experiments, taking into account newly acquired information from the previous runs. It is time-consuming to perform such evolutionary re-formulation of simulations using rather low-level statically typed compiled languages such as C/C++ and Fortran, because of the overheads incurred due to the traditional “write-compile-run” cycle.

In order to resolve this dilemma, many established simulators either include a built-in interpreter of a domain-specific language to describe simulations, or provide bindings to a dynamic language interpreter such as Python. This can then be used to control the core of the simulator, express the models under investigation, and modify them in a much more agile manner. In Section 2.1 we review the approaches adopted by several established neuronal network simulators and in Section 2.2 to 2.4 we elaborate on the direction that we have taken.

### 2.1. Python bindings for a neuronal network simulator

CPython (the reference implementation of Python in C) features a comprehensive C/C++ API^4^ for both *extending* and *embedding* the Python interpreter. This API makes it possible to implement new functions and define objects along with their methods in C/C++ to make them available to the users of the interpreter in form of dynamically loadable modules. Additionally, it makes it possible to build the Python interpreter into an application for use as a scripting language.

In the case that a neuronal network simulator has a well-defined public API, an automatic bindings generator such as SWIG^5^ or Py++^6^ can be employed. The former is exploited in PyMOOSE (Ray and Bhalla, 2008), the Python interface to the MOOSE simulator, while the latter is used in PCSIM (Pecevski et al., 2009) in conjunction with the Boost.Python^7^ library to generate the Python (primary) interface to the PCSIM simulator. The obvious benefit of this technique is that a comprehensive set of bindings covering the entire public API is automatically generated from the original source code with minimal effort on the part of the programmer, based on a rather succinct set of instructions for the bindings generator. The resulting bindings readily allow the software to interact with Python, with the option of bidirectionality: application functions can be invoked from Python and Python callbacks can be registered with the application.

Many existing simulation codes were originally designed as applications rather than libraries. Their user-facing interfaces are the respective command interpreters, which shield the details of the internal class hierarchy and low-level class methods implementations from the user. The NEURON (Carnevale and Hines, 2006) and NEST (Gewaltig and Diesmann, 2007) simulators exemplify this class of applications: NEURON embeds the HOC interpreter (based on the original High Order Calculator by Kernighan and Pike, 1983), and NEST provides SLI (Simulation Language Interpreter; a language that inherits from PostScript by Adobe Systems, Inc., 1999). Both simulators have evolved together with their respective interpreters and the set of established idioms for the interpreter comprises their *de facto* stable high-level public APIs.

When designing an application with a command interpreter, it is still possible and desirable to separate the simulation kernel from the command interpreter, thus defining a clean internal API. This could later be exposed as a public API, to which an automatic bindings generator can be applied. If this separation of concerns is not built in from the outset as a design goal, a number of architectural deficiencies can arise that make it difficult to define an API at a later time. Among such deficiencies are convoluted class hierarchy for object-oriented applications, tight coupling between classes, proliferation of constructs introducing shared state, and insufficient compartmentalization. If a complete refactoring of the code to address these architectural issues is not possible due to limited resources, exploiting the interpreted language as a public API is the only practical option.

The NEURON simulator exposes the state of the HOC interpreter through its Python bindings via an instance of a Python object defined using Python/C API (Hines et al., 2009). It allows this state to be read out and manipulated by means of the normal Python object attribute access.

**Figure 1:**
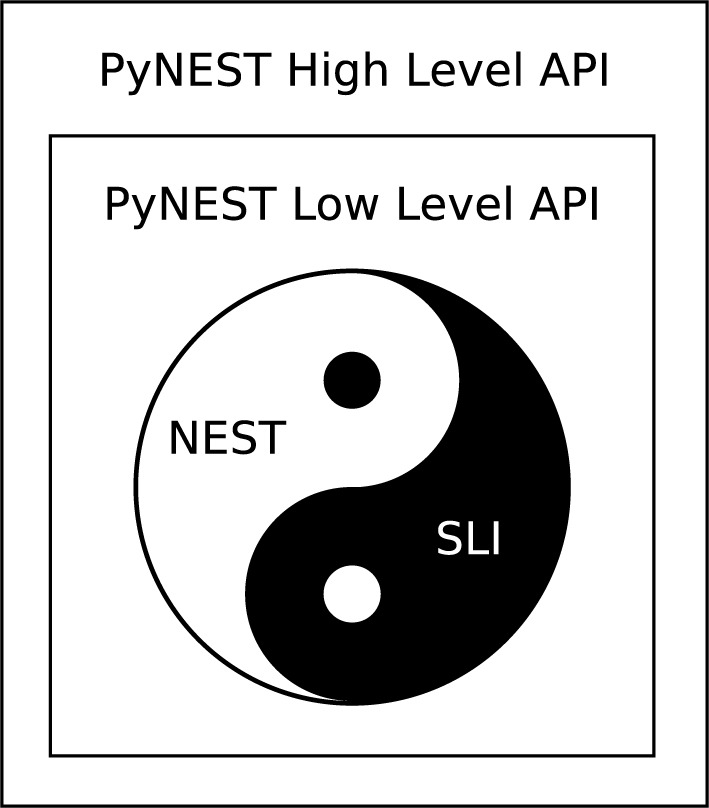
**Diagram depicting the design of PyNEST, the Python bindings for the NEST simulator**. The inner circle represents the NEST simulation kernel and the SLI interpreter. The simulation kernel of NEST uses private SLI data structures (black dot), and many SLI commands are implemented using private NEST simulation kernel APIs (white dot); this non-separation of concerns is discussed in details in the main text. Taken together, however, they effectively constitute a stable high-level public API to the NEST simulator. The first rectangle that encompasses the circle represents PyNEST low-level (private) API, previously implemented in C++ and now re-written in Cython. This API provides three primitives to control the SLI interpreter: a primitive to feed a string as a command for the SLI interpreter to run, and two primitives to push objects on the stack and pop objects off the stack. The outer rectangle illustrates PyNEST high-level (public) API, implemented in pure Python. This high-level API uses the low-level API to provide a “pythonic” interface to the functionality of NEST, such as the creation of neurons, wiring routines, etc.

The overall design of PyNEST (Python bindings for NEST) is depicted in Figure 1; for a detailed sequence diagram showing the interaction between Python and SLI, including the directions of the calls, refer to Figure 2 in Eppler et al. (2008). Due to SLI being a primitive stack machine, a simpler approach than that of NEURON can be applied. PyNEST provides only three low-level primitives to manipulate the SLI interpreter from Python. The first primitive (sli_run) executes SLI code formulated by the high-level API and passed as a string to the SLI interpreter. The other two primitives are concerned with passing data around: sli_push pushes a Python object down the SLI interpreter's stack by converting it to a SLI object and sli_pop pops a SLI object off the SLI interpreter's stack by converting it to a Python object. The high-level NEST Python API is implemented in pure Python and is the one most seen by users. It uses these interpreter manipulation primitives to access the higher level functionality provided by the SLI/NEST command library, e.g., the connection routines employed to wire the neurons, routines to query and set object properties, etc.

Although the low-level NEST Python API that interacts directly with the SLI interpreter (pynestkernel.so) is substantially simpler than the NEURON Python API, it comprised more than 1000 lines of C++ code excluding comments and whitespace (as reported by the cloc utility^8^). The majority of these >1000 code lines contained calls to Python/C API and NumPy/C API. Moreover, much of the rest of them were scattered throughout the source code of SLI, due to an earlier decision to use the visitor pattern to implement the conversion of SLI objects into Python objects. The visitor pattern is a commonly used design in object-oriented programming that separates an algorithm from the entity on which it operates (see Gamma et al., 1994 for a thorough description). For the details on how it was previously used in PyNEST, see (Eppler et al., 2008) and specifically the section “From SLI to Python.”

This turned out to be problematic mainly for two reasons. First, the Python/C API continues to evolve with the new versions of the CPython interpreter, especially during major transitions like Python 2 → Python 3. If one intends to support several versions of the interpreter from the same code base, this by necessity leads to the proliferation of conditional code branches, which imposes additional maintenance burden on NEST developers. The same reasoning applies to the NumPy/C API, which is also subject to change. Second, it introduces a dependency upon the compile-time presence of NumPy development header files on the target machine. This was a frequent cause of confusion: the user would first install NEST and then NumPy, but the latter would not become available to NEST without re-compilation. It was possible to solve this second problem by implementing support for the new-style Python buffer protocol (PEP 3118^9^), however this would have aggravated the situation with respect to the first problem.

### 2.2. Building bridges with Cython

In view of the issues outlined in the previous section, and due to the increasing pressure to support CPython 3, the implementation of the new major revision of the Python language, we were driven to reconsider the original approach of manually implementing the low-level PyNEST API in C++ using Python and NumPy/C APIs. We identified Cython (Behnel et al., 2011) as a tool that not only solved the problems that we had with the previous implementation, but also presented a number of previously unanticipated compelling advantages (see Section 3.1). Since Cython natively supports C++, we opted to completely replace our low-level API code with a Cython implementation. For the remainder of this manuscript, we will refer to the version of the software with the low-level API implemented in Cython as CyNEST; PyNEST refers to the previous implementation with hand-crafted low-level API implementation using Python and NumPy/C APIs.

Cython is the name of both the relevant superset of Python language and the optimizing compiler for this language. The compiler works by generating C or C++ source code (containing Python/C API calls) from Cython code, which can in turn be compiled by an optimizing C/C++ compiler, such as GCC, into a stand-alone application embedding the Python interpreter, or a binary dynamic shared object (DSO). The resulting DSOs can be loaded into Python by importing them from within the Python code, just as any other extension module. Therefore, Cython can in some sense be considered as an abstraction for Python/C API. This raises the question of how stable its interfaces are. So far, the development strategy of Cython has been to minimize backwards-incompatible changes that would impact downstream users. Having worked with Cython for several years, we can attest that this has never been a problem for us in practice. Additionally, it is worth noting that Cython aims to be source-compatible with Python in “pure Python” mode^10^, in which functions provided by the cython module are used to augment the code rather than Cython syntax extensions. A comprehensive discussion of the Cython language and compiler, its advantages and disadvantages, and where it stands in the Python ecosystem is presented in Behnel et al. (2011); an introduction to the language including an example of using Cython for wrapping a C library is given in Behnel et al. (2009).

### 2.3. Porting to Python 3

#### 2.3.1. Background

Python 3, the new major revision of the Python language, offers a large number of enhancements to the syntactic consistency of the language as well as updates to the standard library and performance improvements of the reference implementation. Unfortunately, some of these features required backwards-incompatible changes to be made to Python 3, which considerably slowed down its adoption since its initial release in 2008.

In the specific context of the scientific Python community, the usability of Python is largely determined by the availability of three core libraries: NumPy, which provides a foundation for fast and memory-efficient manipulation of n-dimensional arrays, SciPy, which packages key scientific computing algorithms and functions, and Matplotlib, which provides plotting functionality. These core libraries introduced Python 3 support in 2010, 2011, and 2013, respectively. With these libraries having achieved Python 3 compatibility, it has become attractive to migrate scientific software to take advantage of the cleaner syntax, new features and performance enhancements.

One performance boost that is particularly relevant for NEST is the dramatic reduction in the number of stat() calls issued by the Python import machinery^11^. A stat() call is a standard UNIX system call^12^ that obtains metadata about a named file, such as size, protection mode, creation and modification times, and so on. This reduction was achieved by adding a directory entry cache to the importlib's file finder, with further improvements under way^13^. Since there is a growing drive to use the Python interface of NEST on ever larger clusters, backed by shared network filesystems such as NFS and GPFS, and the speed of metadata operations is a known performance bottleneck for large distributed file systems, minimizing the number of stat() calls is especially important.

Unfortunately, while other groups have observed similar problems with a huge number of stat() calls unacceptably slowing down the startup of the interpreter (Enkovaara et al., 2011), their proposed solutions did not address the root cause. Instead the Python interpreter was modified to use MPI such that metadata operations only happen on one process, and results are broadcasted to other ranks. Since the release of Python 3.3, the aforementioned performance improvements are available to all Python users and do not require applying any modifications to the interpreter or the user code.

The developments discussed above convinced us that the next major release of NEST should fully support Python 3.

#### 2.3.2. Implementation

In order to port Python 2 code to Python 3, an automated translation tool such as 2to3 can be applied to the code base, typically at installation time. The applicability of this approach is limited by the “intelligence” of the translation tools and changes to the code base must often be made to assist the tools to produce correct results. It is also necessary to check the output of the translators, especially after making extensive changes to the code, to ensure that the automatically generated patches do not introduce subtle differences in the logic.

Alternatively, the single source approach can be pursued: the code uses only constructs that work the same way across the major versions of Python, such that exactly the same source runs both on Python 2 and Python 3 interpreters. This might initially sound very limiting, but Python developers have put a great deal of effort into backporting features from Python 3 to Python 2 in a forwards-compatible manner, so that they can also be used in code targeting Python 2 interpreters. For example, the print() function has been available since Python 2.6, and hence using it instead of the print keyword makes it possible for a single source to work on the interpreters of Python 2.6+. Additionally, portability helpers such as six^14^ provide wrappers for functions that have changed names and/or semantics. These wrappers can be used to write code so that it works with both Python 2 and Python 3. Consequently, the single source approach can be an interesting option, although it necessitates adding extra dependencies upon portability helpers or temporarily giving up on some conveniences provided by Python 3.

Finally, some projects that are heavily reliant upon low-level Python implementation details or advanced language features might evaluate the decision to maintain separate branches of code for each major version of Python. This method is seldom put into practice because of the large amount of maintenance work that it entails.

The documentation provided with Python includes a comprehensive porting guide that discusses these possibilities in detail^15^. In order to decide on a technique that would be most appropriate for the Python front-end to NEST, we had to consider two of its layers: the low-level API and the high-level API (Figure 1).

The low-level API, which was previously written in C++, was essentially ported in the process of rewriting it in Cython, because the latter transparently abstracts the differences between Python 2/3. The generated C++ code contains conditional preprocessor definitions that ensure the compatibility of the single source file with all supported versions of Python.

The high-level API was written in pure Python and hence required some adaptation. Having carefully considered NEST usage statistics, we decided to limit the supported Python versions to Python 2.6 and higher. The distributions shipping Python 2.5 have already mostly reached end-of-life, while distributions shipping Python 2.4 are still in use mainly on clusters running Red Hat Enterprise Linux 5 and its derivatives, where newer versions of Python are typically provided to the users via the modules system. Besides, as explained in the porting guide, limiting oneself to Python 2.6 and higher is critical for the feasibility of the single source approach.

In view of these considerations, we opted to start porting from the single source and make the decision on whether to use helpers such as six at a later stage. As it turned out, after updating the code to use Python 2.7 best practices, there remained only one instance in which conditional branching depending on Python 2/3 was inevitable. Thanks to the substantial test coverage (Eppler et al., 2009) and a large library of examples, the remaining Python 3 porting effort basically consisted of identifying the underlying reasons for test failures and fixing the code to use the idioms accepted by both Python 2/3 interpreters. A responsive continuous integration system (Zaytsev and Morrison, 2013) giving rapid feedback on the number of tests still failing and the amount of broken example code supported this process considerably.

### 2.4. Alteration of build system

A long-standing grief with PyNEST was the state of its build system: since pynestkernel.SO includes full-fledged copies of the NEST simulation kernel and the SLI interpreter, its compilation by necessity required re-building a large complex C++ code base composed of hundreds of files, which is otherwise built with Autotools and Libtool (Calcote, 2010). Originally implemented with distutils^16^, it quickly became arcane and later completely unmaintainable due to the amount of custom hooks required to replicate the functionality of Autotools. In addition, these hooks proved to be rather fragile, because they relied upon parsing temporary files generated by the main build sequence.

During the rewrite (which could have also been implemented for the original system), it was decided to leave the building of the shared object from the C++ source generated with Cython to Autotools, using distutils only to install pure Python code and inject a binary shared module built with Autotools as a payload. An additional noteworthy advantage of this strategy is in that it enables the Python bindings to be cross-compiled using the standard mechanisms available in Autotools. Whereas it is not impossible to hijack distutils classes to use a different toolchain from the one used to compile the host interpreter, this solution is very complex and hard to maintain. With Autotools, the only required extra piece of information to be supplied is the location of the headers of the target interpreter, as they cannot be automatically detected; the rest is taken care of automatically.

## 3. Results

### 3.1. Maintainability of code

The new implementation of the low-level API consists of only two files: pynestkernel.pyx, which contains Cython source code and pynestkernel.pxd, which contains definitions, similar to C/C++ header files. The original implementation in C/C++ consisted of 7 core files and touched 22 other files from the SLI interpreter source code. The affected files contained the definitions of SLI data types, and previously had to be modified to add calls to the converter class used to coerce them to the appropriate Python data types. Additionally, the previous strategy required splitting several files into interface and implementation parts to break cyclic dependencies, which is no longer necessary for the new implementation.

The new code is not only more straightforward, being written in a higher level language, but also contains no Python/C API calls. Behavioral differences between Python versions and API changes are all transparently handled by Cython behind the scenes. It is also fully self-contained, so all modifications made to the SLI interpreter are no longer necessary and were reverted. Moreover, it is half the size of the previous implementation, containing <500 lines of code according to the cloc utility, about ~30% of which consisting of trivial definitions.

Furthermore, thanks to the “Typed Memoryviews”^17^ feature of Cython, we can now process *any* Python objects providing PEP 3118 buffers transparently (including native array.array objects), along with NumPy 1.5.0+ arrays using a clear and concise syntax devoid of any additional branching. This requires neither a compile-time dependency on NumPy, nor manual handling of strides, which is rather difficult to get right and is known to have caused problems in PyNEST in the past.

The reduction of the source code footprint and the use of easier to understand constructs, which are thus more maintainable, are the most noticeable benefits of the rewrite of the low-level PyNEST API (Figure 1) in Cython as described in detail in Section 2.2. They can be generally attributed to a greater expressiveness of higher level languages as compared to lower-level languages, and hence are unsurprising. However, we also observed a number of advantages, which are not immediately obvious, but are nevertheless important for the maintainability and the correctness of the code:

1. Since Cython abstracts the differences between Python 2/3, we now have the benefits of working on a single source without any version-dependent branches.
2. Manual balancing of the Python object reference counts, which is notoriously hard to handle^18^, is no longer an issue with Cython due to the “reference nanny” it provides to verify generated code.
3. Exceptions thrown on the C++ level are correctly propagated to the level of Python, and no longer abort the interpreter, thanks to the in-built support for the “except+” clause by Cython.
4. Correct handling of Unicode strings with Cython requires no additional effort from developers, due to the string handling (encoding and decoding) functions it makes available ^19^.

The concept of the reference nanny requires some additional explanation. CPython primarily uses reference counting garbage collection for automatic memory management, which works by keeping track of the number of times an object is referenced by other interpreter objects. The reference count is incremented whenever a new reference to the object is created, and decremented when a reference to the object is removed. If the reference count goes to zero, the object is identified as garbage and deallocated. From the point of view of the Python/C API, reference counting is implemented via the py_incref()/py_decref() macros and it is the responsibility of the programmer to ensure the right balancing.

In order to assist programmers in finding memory management errors such as memory leaks or premature deallocations, Cython's reference nanny provides special versions of these macros, which are used in the generated code to record the number of times the corresponding C API macros have been called. If the reference nanny is inactive, these special macros simply become aliases to the corresponding original macros. The reference nanny is enabled by default for Cython's own test suite and has to be manually activated with a preprocessor directive for user code.

Note that this is not necessarily applicable to other Python implementations, such as PyPy, which comes with a tracing garbage collector and does not rely on reference counting. However, alternative implementations have to emulate said macros for C API level compatibility with CPython, and hence it is still important to have them right.

To summarize, the Cython features discussed in this section facilitate the reduction of the amount of extension code, its complexity and the number of its compile time dependencies.

### 3.2. Maintainability of the build system

The re-write of the build system described in Section 2.4 not only reduces the amount of lines of code by a factor of two (according to the cloc utility report), but also addresses many other enduring problems, such as unreliable installation path detection on multiarch platforms and inconsistent incremental builds due to flawed dependency calculation. We can only recommend investigating this approach to other developers in a similar situation.

The build system revision constitutes a typical example of counter-revolutionary kind of maintenance as defined in the Introduction. Even though the previous version of the build system was in working order (with the exception of the abovementioned known issues), the assumptions that were made at the time of the original implementation turned out to be incorrect. Indeed, a purely distutils-based approach has proven to be fragile and difficult to maintain. Hence, continued maintenance of this suboptimal implementation can be regarded as interest on the accrued technical debt, which is now paid back by updating the system based on a more accurate set of assumptions.

It is important to note that these changes did not cause any degradation in user experience. In spite of the introduced separation of concerns between Autotools and distutils, the build and installation processes are still launched by “make” and “make install” commands respectively. Furthermore, the use of Cython by the developers does not impose an additional compile time dependency on the users. Future releases of NEST will ship with a pre-compiled version of CyNEST low-level API implementation in form of a regular C++ file (along with the pyx/pxd sources), which will be used during the build process. Only developers wishing to bootstrap the build system from scratch for the pre-release versions and/or make custom modifications to the Cython code will need to have the Cython compiler installed on their systems.

#### 3.2.1. Cross-compiling CyNEST

Owing to the inclusion of the patches enabling cross-compilation in the stock distribution since Python 3.3, we were able to cross-compile Python 3.4.0b2 for the IBM BlueGene/Q supercomputer JUQUEEN installed at the Jülich Research Center, Germany with only minor changes. Detailed performance and scalability assessments are subjects of current research, and work is still in progress on the systematic testing of the resulting build and the inclusion of patches resolving identified issues upstream. However, preliminary scaling tests up to the full machine (458,752 cores/1,835,008 threads) have confirmed that the improvements to the module loader described in Section 2.3.1 indeed allow the interpreter to be initialized and an MPI communicator to be established in less than 3 min, which makes it practicable to use Python on this scale.

As a proof of concept, we were able to cross-compile CyNEST for this interpreter and run a test simulation on one BG/Q nodeboard (2048 threads); the required changes to the build system will be part of future NEST releases. This is the first recorded instance of running a cross-compiled Python front-end to NEST that we are aware of.

### 3.3. Performance and scalability

There were various design decisions made during the development of the Cython implementation of the low-level API of the Python front-end to NEST that could potentially reduce the performance or the scalability of NEST. Firstly, we abandoned the visitor pattern for type conversion (see Eppler et al., 2008) and restructured the code by breaking large functions into smaller ones, thus increasing the function call overhead. Second, the Cython features of C++ exception propagation and proper handling of strings (see Section 3.1) incur additional costs.

To investigate whether our Cython implementation gives rise to a significant reduction in performance or scalability, we ran a set of synthetic benchmarks on PyNEST and CyNEST low-level API primitives. These benchmarks consisted of repeatedly calling the PyNEST/CyNEST primitive under investigation to measure its runtime, and were performed on a KVM virtual machine running a minimal installation of Fedora Core 18 i386 operating system (shipping Python 2.7.3 and Python 3.3.0), assigned one core of the Intel Xeon X5680 CPU @ 3.33 GHz and 8 Gb of RAM. We used Python 2.7.3 for all of our tests, because the legacy PyNEST implementation does not support Python 3. The Python timeit module was used to call each of the primitives at least 10^7^ times with at least 5 repetitions. For sli_push() and sli_pop() primitives, the interpreter stack was prepared in advance to contain a sufficient amount of elements, in order to minimize interference from non-relevant code which we would otherwise have needed to insert inside the measurement loop. The best timing was selected for each experiment.

The results are presented in Table 1 and demonstrate that CyNEST primitives are currently slower than PyNEST by 30–150%. This slowdown can be explained by the function call overhead, exception handling and (previously non–existent) null pointer checks. However, these synthetic benchmarks are not representative for real use cases. In most realistic situations, the time taken for network construction, simulation and analysis dominates the total runtime, with the time taken for the Python bindings to communicate with the NEST kernel contributing only a small fraction.

**Table 1:** **Comparison of execution timings of low-level API primitives in PyNEST and CyNEST**.

**LL-API call**	**PyNEST [μS]**	**CyNEST [μS]**
sli_run()	18	24
sli_push()	0.25	0.5
sli_pop()	0.15	0.4

*For the sli_run() primitive the command “clear” was used as a parameter, but the results were similar for an empty command string. For the sli_push() and sli_pop() primitives, an arbitrarily selected integer *x* = 42 was chosen as a test value. PyNEST (the original implementation) was benchmarked at the last revision in the Subversion repository before the merge (r10626), CyNEST (the new implementation) was benchmarked at r10683. These revisions correspond to the development versions of the code which will eventually supersede the current publicly available release of NEST 2.2.2*.

Therefore, in order to get an insight into how such a slowdown would affect real-world simulations, as opposed to our synthetic benchmarks, we selected a simplified version of the Hill-Tononi model of the early visual pathway (Hill and Tononi, 2005), implemented using the NEST Topology module (shipped with the public releases of NEST as “hill_tononi_Vp.py”). The total model runtime under CyNEST includes contributions from the new low-level API kernel implemented in Cython, as well as from substantial modifications to the high-level API to clean up its internals and add support for Python 3. The replacement of the low-level API implementation written in C++ with the new one written in Cython required an extensive adaption of the high-level API, and thus it is not possible to measure the impact directly by simply exchanging the binary kernels. Instead, we traced the execution of the model, measured the number of calls to the low-level API primitives, and computed the predicted total impact, caused by the changes to the low-level API.

The breakdown is shown in Table 2 and indicates that the predicted increase of the runtime due to the low-level API changes is of the order of magnitude of 0.2 s, which is a negligible proportion of the total runtime of the simulation on the hardware and software setup described above. Additionally, we measured the total runtime of the model using PyNEST and CyNEST presented in Table 3 and observed an overall slowdown of ~1.3 s, which we attribute largely to the changes in the high-level API, such as more extensive parameter checks and usage of SLI literals wrapping Unicode strings as dictionary keys instead of binary strings. We conclude that the observed performance degradation is a tiny price to pay for the benefits outlined in the previous sections and does not negatively affect any real simulations carried out with NEST.

**Table 2:** **Simplified Hill-Tononi model as a benchmark for PyNEST and CyNEST**.

**LL-API call**	**Invocations**	**Slowdown [μS]**	**Impact [s]**
sli_run()	38947	6	0.2
sli_push()	40484	0.25	0.01
sli_pop()	74497	0.25	0.02

*The table contains the number of invocations of each of the three low-level API primitives, slowdown per invocation per primitive (see Table 1), and the corresponding predicted total impact*.

**Table 3:** **The runtime and memory consumption measurements performed on the untraced version of the simplified Hill-Tononi model using single-threaded simulation on the hardware/software setup described in the main text**.

	**Median**	**Minimum**	**Maximum**
**RUNTIME OF THE MODEL [s]**			
PyNEST	64.8	63.8	66.9
CyNEST	66.1	65.5	71.6
**PEAK MEMORY USAGE [MiB]**			
PyNEST	327	327	328
CyNEST	329	329	329

*The runtime (which includes the collection of the results and the production of the graphics) was obtained using the shell time command and the memory usage was recorded using Massif. Each experiment was repeated *n* = 15 times, alternating the measurements for PyNEST and CyNEST*.

Since NEST with a Python front-end scales in the same way as NEST with its native SLI front-end, the scalability is currently limited by its memory consumption (Kunkel et al., 2012). If the new implementation required substantially more memory than the original implementation, it would thus compromise the scalability of NEST. In order to assess the memory consumption of the re-written extension in real-world simulations, we measured the memory consumption of the previously described simplified Hill-Tononi model using Massif heap profiler, which is a part of the Valgrind ^20^ instrumentation framework.

As shown in Table 3, we were unable to detect any significant differences: the discrepancy in peak memory usage between PyNEST and CyNEST amounted to <1%. In addition to that, a synthetic benchmark that consisted of running the getconnections() function on a network with at least 10^7^ connections and measuring the memory consumption of the process, showed ~15% improvement in memory usage. We attribute this improvement to the smaller overhead of Python arrays in CyNEST as compared to NumPy arrays in PyNEST for the small number of elements used to represent the connection objects. Therefore, we conclude that the rewrite in Cython does not negatively affect the memory consumption and hence does not degrade the scalability of NEST.

## 4. Discussion

The outstanding productivity that can be achieved by researchers using the Python bindings for NEST to set up their simulations is one of the key factors in its continued success as a research tool for simulating large networks of spiking point neurons or neurons with a small number of electrical compartments. This success, however, comes at the cost of reduced maintainability of the code base, in large part due to the difficulties in simultaneously supporting several versions of Python.

The reduction in maintainability is due to the architecture of NEST. For software applications that provide a stable well-defined public API, it is often possible to make use of automatic bindings generators such as SWIG or XDress ^21^, and a number of simulators such as PCSIM and MOOSE have benefited from this approach in the past, as described in Section 2.1. In the case of NEURON and NEST, this technique turned out to be impractical. Consequently, the Python bindings were originally written by hand using Python/C API, which leads to the maintainability issues mentioned and increases a project's technical debt.

We therefore investigated an alternative approach, namely implementing the low-level Python API of NEST in Cython, a superset of Python (see Section 2.2), which can be automatically compiled into C/C++ code. We also carried out a set of related measures to improve the maintainability of the code base, specifically porting to Python 3 through the single source technique and re-designing the build system.

This approach resulted in a reduction of the code footprint of around 50% and a significant increase in the cohesiveness of the code related to the Python bindings: whereas previously seven core files and 22 additional files were involved, the new approach requires merely two core files. The new implementation also removes the compile-time dependency on NumPy and provides numerous additional maintainability benefits by reducing complexity and increasing comprehensibility of the code. The re-write of the build system also resulted in a 50% reduction of code, and resolved multiple issues with its usability and robustness. We analyzed the performance of the new implementation, and discovered that although the low-level API calls in CyNEST are more costly than in PyNEST, the difference is only detectable in carefully designed synthetic benchmarks. For all sensible use cases, the extra overhead is negligible. Additionally, the new implementation decreases the memory footprint, so that the scalability of CyNEST is not deteriorated with respect to PyNEST or NEST using the native SLI front-end.

We can recommend this approach for many other projects in which a Python front-end is required to bind with C or C++ functions. The use of languages other than C/C++ does not necessarily preclude taking advantage of Cython, provided that the language in question has a good foreign function interface (FFI) for C, such as Fortran. The possibility of creating more “pythonic” bindings by exposing an API that operates with data structures commonly used in Python (for instance, functions returning lists, or consuming iterators) and seamlessly blends with idiomatic Python code, can be an additional benefit of hand-crafting bindings in Cython, although not one that was exploited in the current study. For applications written in Java, it would typically be more appropriate to use Jython^22^, which provides a seamless integration with Java Virtual Machine based languages. One advantage of NEST with respect to our approach is that it only has three low-level API primitives. For software applications with a larger number of such calls, the re-implementation of the Python bindings in Cython would naturally involve somewhat more work, but this is largely of a trivial nature. Each API call requires one function definition in the PXD file (one line) and one wrapper function in Cython that calls the declared C-function (two or more lines).

We would not recommend our approach for a software project which has sufficient resources to re-design the architecture such that the interpreter is separated from the computation kernel. In this case, the re-design would be viewed as the ideal approach. Not only does this permit the use of automatic bindings generators, but it also reduces coupling, increases cohesiveness and permits greater testability. Finally, the architectural improvements do not preclude a combined approach, where an additional layer written in Cython and/or pure Python is provided on top of the automatically generated bindings in order to improve the API, as mentioned above.

The current study demonstrates that efforts to reduce the technical debt of a project can have far-reaching consequences beyond the original goals. As mentioned in Section 2.3.1, the latest advances in bringing Python to HPC gave us the incentive to reconsider the previously established notion that it is impractical to use the Python front-end of NEST on supercomputers. The remaining challenge was in the cross-compilation of the binary extension module for the compute nodes (where the code is run), which generally provide a different environment from that of the front-end nodes (where the code is compiled). Since the compilation of CyNEST is now managed by Autotools (see Section 2.4) and NEST with the SLI front-end could already be cross-compiled using Autotools for the compute nodes, no fundamental obstacles remained to cross-compilation. We were able to work around the few remaining issues with the build system and cross-compile the Python front-end for NEST for the IBM BlueGene/Q supercomputer; these changes will be included in future releases of NEST. As a result of these changes, researchers working on supercomcomputers can benefit from the same advantages in describing and controlling simulations as are currently available for personal workstations and small clusters. Moreover, a researcher can now directly scale up simulations coded in Python and developed on personal workstations or laptops to big machines without first translating them into SLI, which was previously the only available option. It is important to note that supercomputers are not the only targets that require cross-compilation: at the other end of the computing spectrum, cross-compiling is required for most widely available embedded platforms. Therefore, the ability to cross-compile also opens up new possibilities for using NEST with a Python front-end on embedded systems, e.g., for robotics and control applications.

The re-implementation in Cython also makes it possible to use the resulting extension with other implementations of Python than the official CPython implementation at practically no cost, provided that support for those implementations is added to the Cython code-generating back-end. One such implementation of particular interest is PyPy^23^, which features a Just-in-Time (JIT) compiler that potentially allows Python code to run not only faster than interpreted by CPython, but faster even than hand-crafted code written in compiled languages, such as C/C++ or Fortran. Therefore, complex pre-processing of the data required to set up neural network simulations, or post-processing of simulation results could be implemented in pure Python, taking full advantage of its dynamic nature, and run using the same interpreter. A feasibility study has been performed in the scope of this project, and a working prototype of CyNEST that runs on PyPy has been created. However, it is not yet possible to achieve an “out of the box” translation that would work without manual changes to the generated code, due to the current imperfect interoperability of Cython with PyPy. Another implementation worth mentioning is Jython, which might get a Python/C API emulation layer that can be exploited by Cython as the JyNI^24^ project matures. This implementation is interesting, because it allows one to profit from the performance of JVM, and enables seamless integration with JVM-based languages which are especially popular in the industry. Currently, JyNI is at the early “alpha” stage, so it is not yet stable enough to be considered as a potentially supported target for Cython.

In conclusion, we hope that through a more widespread use of Cython, neuroscientific software developers will be able to focus their creative energy on refining their algorithms and implementing new features, instead of working to pay off the interest on the accumulating technical debt.

### Conflict of interest statement

The authors declare that the research was conducted in the absence of any commercial or financial relationships that could be construed as a potential conflict of interest.

## Footnotes

^1^http://brainscales.kip.uni-heidelberg.de

^2^https://www.humanbrainproject.eu

^3^Source code abundant in complex and entangled control structures, especially branching, that encumber comprehension of the program flow.

^4^http://docs.python.org/c-api/

^5^http://www.swig.org

^6^http://sourceforge.net/projects/pygccxml/

^7^http://www.boost.org/libs/python/

^8^http://cloc.sourceforge.net

^9^http://www.python.org/dev/peps/pep-3118/

^10^http://docs.cython.org/src/tutorial/pure.html

^11^http://bugs.python.org/issue14043

^12^http://pubs.opengroup.org/onlinepubs/009695399/functions/stat.html

^13^http://bugs.python.org/issue18810

^14^https://pypi.python.org/pypi/six/

^15^http://docs.python.org/3/howto/pyporting.html

^16^http://docs.python.org/distutils/

^17^http://docs.cython.org/src/userguide/memoryviews.html

^18^Refer, for instance, to the cpychecker project by David Malcolm of Red Hat, Inc., which helped to identify a large number of programming mistakes of this kind: http://gcc-python-plugin.readthedocs.org/en/latest/cpychecker.html

^19^http://docs.cython.org/src/tutorial/strings.html

^20^http://valgrind.org

^21^http://xdress.org

^22^http://www.jython.org

^23^http://pypy.org

^24^http://jyni.org
